# NMDA receptor-dependent long-term potentiation comprises a family of temporally overlapping forms of synaptic plasticity that are induced by different patterns of stimulation

**DOI:** 10.1098/rstb.2013.0131

**Published:** 2014-01-05

**Authors:** Pojeong Park, Arturas Volianskis, Thomas M. Sanderson, Zuner A. Bortolotto, David E. Jane, Min Zhuo, Bong-Kiun Kaang, Graham L. Collingridge

**Affiliations:** 1 Department of Brain and Cognitive Sciences, College of Natural Sciences, Seoul National University, Seoul 151-746, South Korea; 2 Centre for Synaptic Plasticity, School of Physiology and Pharmacology, Dorothy Hodgkin Building, Whitson St., Bristol BS1 3NY, UK; 3 Department of Physiology, Faculty of Medicine, University of Toronto, 1 King's College Circle, Toronto, Ontario, CanadaM5S 1A8; 4 Department of Biological Sciences, College of Natural Sciences, Seoul National University, Seoul 151-746, South Korea

**Keywords:** NMDA receptor, AMPA receptor, long-term potentiation, short-term potentiation, hippocampus, learning and memory

## Abstract

*N*-methyl-d-aspartate receptor (NMDAR)-dependent long-term potentiation (LTP) is extensively studied since it is believed to use the same molecular mechanisms that are required for many forms of learning and memory. Unfortunately, many controversies exist, not least the seemingly simple issue concerning the locus of expression of LTP. Here, we review our recent work and some of the extensive literature on this topic and present new data that collectively suggest that LTP can be explained, during its first few hours, by the coexistence of at least three mechanistically distinct processes that are all triggered by the synaptic activation of NMDARs.

## 1. Introduction

Long-term potentiation (LTP) and its counterpart long-term depression (LTD) are the major forms of long-lasting synaptic plasticity in the vertebrate central nervous system (CNS). As described in other articles in this issue, LTP and LTD are the probable substrates for many forms of learning and memory [1] and their dysregulation probably contributes to a wide diversity of brain disorders [2–5]. LTP was first described at the perforant path synapse [6,7], a powerful monosynaptic excitatory projection from entorhinal cortex to the dentate gyrus of the hippocampal formation. LTP has since been described in numerous excitatory pathways in the CNS, including many connections within the hippocampus. In particular, LTP has been extensively studied in the Schaffer collateral-commissural pathway (SCCP), a monosynaptic connection between CA3 and CA1 pyramidal neurons of the hippocampus. At this pathway, using the competitive *N*-methyl-d-aspartate receptor (NMDAR) antagonist 2-amino-5-phosphonopentanoate (AP5) [8], it was observed that LTP is triggered by the synaptic activation of NMDARs [9]. Subsequently, it has been found that NMDAR-dependent LTP (NMDAR-LTP) is the predominant form of synaptic plasticity in the brain [10]. Despite its importance there is still a lot that is not understood about LTP and many controversies exist, for example, regarding the relative roles of presynaptic and postsynaptic mechanisms in the expression of LTP [11–13].

In this article, we present evidence that, at the SCCP, LTP comprises at least three distinct forms of synaptic plasticity that can coexist. We posit that the existence of multiple forms of NMDAR-LTP explains many of the controversies that have raged for far too long.

## 2. Short-term potentiation is a distinct form of synaptic plasticity

LTP was first observed, and is still most commonly studied, following the delivery of one or a few periods of high-frequency stimulation (HFS), delivered either as simple trains (often referred to as a tetanus) or as more complex patterns of theta burst stimulation (TBS). Typically, there is an initial large increase in the response size that decays, usually over a period of tens of minutes, to a stable increase that then persists, generally for as long as recordings are maintained. The initial decremental potentiation is usually referred to as short-term potentiation (STP). The extent to which global potentiation involves an STP component varies considerably between experiments, and in some cases little or no STP is evident. The absence of STP has led some investigators to dismiss STP as irrelevant or, when it occurs, to view it as an unstable initial phase of LTP. We would argue the contrary: that STP is an important and mechanistically distinct part of the potentiation process.

**Figure 1.**
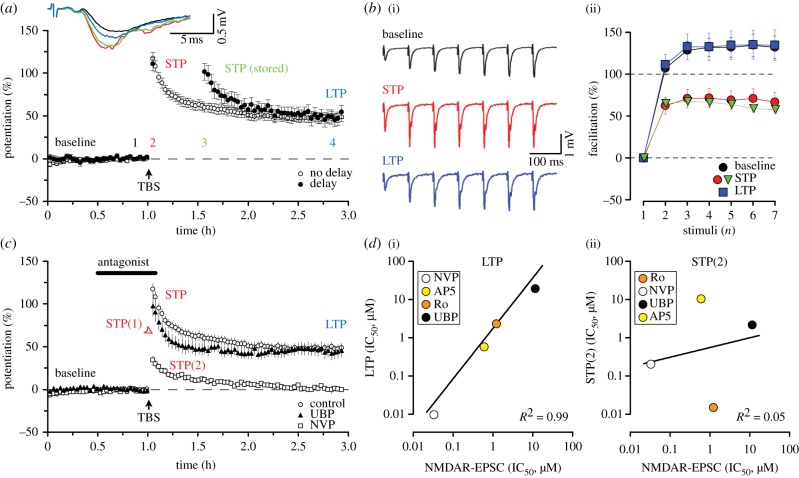
Different NMDAR subtypes mediate the induction of LTP and two forms of STP. (*a*) Pooled data to show LTP in response to TBS (four pulses at 100 Hz repeated 10 times at 5 Hz) under standard conditions (open circles) and with a 30 min pause in stimulation (filled circles) starting after the first four synaptic responses had been obtained (to assess the maximal level of NMDAR-dependent potentiation). Representative traces were obtained at the times indicated by the corresponding coloured numbers. (*b*(i)) Responses to a train of seven stimuli delivered at 12.5 Hz. (*b*(ii)) The graph plots the slope of each response normalized to the first response in the train to show depression in facilitation during either STP (red) or stored STP (green) but not LTP (blue) when compared with facilitation in the baseline (black). (*c*) Effects of NVP (0.1 µM) or UBP (10 µM) on STP and LTP. Note that UBP selectively blocks STP(2) whereas NVP selectively blocks LTP and STP(1). (*d*) The graphs plot the IC_50_ for inhibition of LTP (*d*(i)) or STP(2) (*d*(ii)) versus the IC_50_ for inhibition of the NMDAR-EPSC. Note the excellent correlation for LTP but lack of correlation for STP(2). Insets indicate the antagonists, shown in descending rank order of potency for inhibition of the form of synaptic plasticity studied. Adapted (with permission) from [15] (*a*,*c* and *d*) and [16] (*b*), respectively.

STP was found to have some remarkable properties that distinguish it from conventional LTP and explain its variable appearance, or even lack of appearance, among studies [14]. First, the magnitude of STP depends on the frequency of the HFS that is used to trigger synaptic plasticity; the higher the frequency the greater the magnitude. Second, the duration of STP is determined by two factors: (i) the number of stimuli that are used during HFS (the larger the number the slower its decay) and (ii) the frequency of low-frequency stimuli that are delivered following its induction (the lower the frequency the slower its decay). For example, STP induced by 40 stimuli decays to reach a steady-state level of potentiation after the delivery of approximately 160 stimuli. Consequently, when constant low-frequency stimulation is employed to monitor the level of synaptic plasticity (which is standard practice) then the decline of STP is dependent on the frequency of that stimulation. Therefore, a high-frequency induction protocol favours STP and the lower the frequency of stimuli used to monitor the potentiation the longer it lasts. Taken to an extreme, if stimulation is paused then STP does not decay until the stimulation recommences (figure 1*a*). Furthermore, the use of a Ca^2+^-free solution to prevent neurotransmitter release also prevents the decay of STP, whereas blockage of excitatory amino acid receptors does not. On the other hand, when short high-frequency bursts are given, instead of low-frequency stimulation, to monitor the plasticity, STP does not decay, being maintained at a steady and maximal level until it is probed again at lower frequency. Therefore, STP has the potential to last a long time, and to reflect this property it has also been termed transient-LTP (t-LTP) [14].

Significantly, a pairing protocol, which typically involves a steady intermediate frequency to induce and to monitor potentiation, mitigates against STP and explains why this component is small or absent in many ‘pairing experiments’. Although we acknowledge that STP does not accurately describe a phenomenon that has the capacity to last a long time, we shall retain the name here to describe the decaying component of potentiation that is commonly observed following HFS or TBS.

It has been argued that STP is an unstable component of the LTP process, but is otherwise mechanistically identical to the latter phases. In other words, only in a proportion of the synapses that undergo a modification is the potentiation persistent, in the others it is unstable. However, there are several lines of evidence to suggest that STP and LTP are mechanistically different phenomena. Interestingly, in the first study to employ paired-pulse stimulation to assess the locus of expression of LTP it was found that, in the lateral perforant path *in vivo*, there was no alteration in paired-pulse facilitation (PPF) during LTP [17]. However, an early decaying component of the response was associated with a decrease in PPF. Although the initial potentiation was considered an extension of post-tetanic potentiation it had the appearance of what, by current terminology, would be classified as STP. The difference between STP and LTP with respect to changes in PPF was also clearly observed at CA1 synapses [14]. STP, whether monitored within a few minutes of the TBS or after a pause in stimulation of 30 min, was associated with a substantial change in PPF whereas the steady-state potentiation was not. This same difference was also observed when brief high-frequency bursts were used to monitor the level of potentiation (figure 1*b*) [16]. The simplest interpretation of these findings is that STP is expressed by an alteration in the probability of transmitter release, P(r), whereas LTP is expressed by a postsynaptic modification. This is the same conclusion that was reached when the postsynaptic sensitivity of neurons was assessed by the local application of α-amino-3-hydroxy-5-methyl-4-isoxazolepropionic acid-receptor ligands [18]. There was no change in responsiveness during the STP phase but an increase in sensitivity developed as STP decayed (and hence the stable component of LTP was developing). The increase in AMPAR sensitivity was dependent on the synaptic activation of NMDARs and was sufficient in magnitude to account for the steady-state level of LTP that was observed. Of course, PPF changes and sensitivity changes could have other, less obvious, explanations. For example, in principle, a change in PPF can be explained by a postsynaptic change [19], but rarely has this been the case. Nonetheless, the existence of two distinct functional phenotypes (PPF changes, no alteration in sensitivity versus no PPF changes, alteration in sensitivity) argues strongly that STP and LTP are two temporally and mechanistically distinct processes.

## 3. Different *N*-methyl-d-aspartate receptor subtypes mediate the induction of short- and long-term potentiation

More recently, we have found evidence that STP and LTP can also be separated on the basis of their induction properties. Using a range of subtype-preferring NMDAR-antagonists, we could clearly distinguish different components of synaptic potentiation on the basis of the NMDAR subunits involved in their induction [15]. We characterized the selectivity of the compounds on recombinant receptors (GluN1 co-expressed in HEK293 cells with one of the three GluN2 subunits—GluN2A, GluN2B or GluN2D—that are expressed in the hippocampus) and on pharmacologically isolated NMDAR-dependent excitatory postsynaptic currents (NMDAR-EPSCs). We also constructed full concentration–response curves for the inhibition of STP and LTP. The antagonists that we characterized (and their selectivity) were as follows: D-AP5 (GluN2A = GluN2B > GluN2D) [8], NVP-AAM007 (NVP: GluN2A > GluN2D > GluN2B) [20], Ro 25–6981 (Ro: GluN2B >> GluN2A >>> GluN2D) [21]) and UBP145 (UBP: GluN2D > GluN2A = GluN2B) [22]). The effects of two of these antagonists, NVP and UBP, are illustrated in figure 1*c* [15].

Based on a comparative and detailed concentration–response analysis of the sensitivity of the different temporal phases of potentiation to these antagonists, we concluded that there are two pharmacologically distinct forms of STP, which we have termed STP(1) and STP(2), and one form of LTP (figure 1*c*). STP(2), which was the major contributor to the total STP, had a very distinct pharmacological profile from the other two components of synaptic plasticity, which were similar to one another. For STP(1) and LTP the rank order of potency was as follows: NVP > AP5 > Ro > UBP (figure 1*d*(i)), whereas for STP(2) the rank order of potency was Ro > NVP > UBP > AP5 (figure 1*d*(ii)). Irrespective of the pharmacological specificity of the antagonists used (which is far from perfect and discussed below), we can state with certainty that STP(2) represents a distinct phenomenon from STP(1) and LTP based on sensitivity to the antagonists.

By use of these compounds, we could pharmacologically separate STP(2) and STP(1), the latter together with LTP, sufficiently to study their properties in isolation. We found that STP(2) decayed more slowly than STP(1) when the test stimuli were delivered at the same rate (figure 1*c*). For example, at a test frequency of 0.067 Hz (i.e. 1 stimulus every 15 s) STP(1) decayed with a single exponential time-constant (*t*_D_) of around 7 min whereas STP(2) decayed with a *t*_D_ of around 16 min. Consequently, STP(2) contributes more to the decay of the global STP, while STP(1) contributes more to the peak of potentiation. Interestingly, both forms of STP can be stored in time when the test stimulation is paused [15].

We also compared the ability of the four antagonists to inhibit STP and LTP with their ability to inhibit the NMDAR-EPSC (from the same type of adult slices as used in the plasticity experiments; figure 1*d*). Unsurprisingly, there was a very strong correlation between the inhibition of LTP and antagonism of the NMDAR-EPSC (figure 1*d*(i)). Antagonism of STP(1) also correlated with inhibition of the NMDAR-EPSC. In stark contrast, STP(2) did not correlate at all (figure 1*d*(ii)). These observations are entirely consistent with STP(1) and LTP being induced by the synaptic activation of the same NMDARs that mediate the NMDAR-EPSC, as is widely assumed to be the case [10,23]. However, they suggest that STP(2) is not mediated by these receptors, but rather by NMDARs that do not contribute, at least to any appreciable extent, to the synaptic response. In other words, the NMDARs that mediate induction of STP(2) are probably located either at an extrasynaptic site on the postsynaptic neuron, on glial cells or on presynaptic elements.

Although the NMDAR-antagonists are not ideal pharmacological tools, the comparative and quantitative analysis that we performed has enabled us to deduce the identity of the NMDAR subunits that are responsible for the induction of LTP and STP in adult animals. The role of NMDAR subtypes in LTP (and LTD) has been the subject of intense investigation and is highly controversial. This topic is reviewed in another article in this volume [24].

In terms of LTP, its induction requires the activation of both GluN2A- and GluN2B-containing NMDARs. LTP was highly sensitive to NVP and AP5 at concentrations that were selective towards the GluN2A subunit (figure 1*c*). LTP was also sensitive to Ro. However, relatively high concentrations of Ro were needed to inhibit LTP, a sensitivity profile that suggests that the NMDAR subtype responsible might be a GluN2B-containing triheteromer. (These are known to be less sensitive to ifenprodil analogues than GluN2B-containing diheteromers [25].) Based on the sensitivity to NVP and Ro, and on the analysis of the effects of these antagonists on the kinetics of the NMDAR-EPSC, we concluded that induction of LTP is mediated by the activation of both GluN2A diheteromers and GluN2A/GluN2B triheteromers, with the latter species forming the majority of the receptors.

In contrast to LTP, STP(2) was highly sensitive to Ro, indicative of an involvement of GluN2B-containing diheteromers. It was also sensitive to UBP at a concentration that was selective at GluN2D-containing NMDARs. These observations imply either an obligatory role of both GluN2B and GluN2D diheteromers or, more simply, the involvement of a GluN2B/GluN2D triheteromer (though this would necessitate that this subunit combination retains a high sensitivity to Ro). With respect to STP(1) we cannot say, on the basis of the use of available antagonists, whether it has a similar or an identical pharmacology as LTP. If the latter was the case, then STP(1) could be an early decremental form of LTP, consistent with some suggestions in the literature for STP in general.

In summary, we have been able to subdivide STP into two components, STP(1) and STP(2), on the basis of pharmacological and kinetic criteria. STP(1) and LTP have similar sensitivity to NMDAR-antagonists and could potentially be induced by the same NMDAR subtypes whereas STP(2) is a pharmacologically distinct process, which differs from STP(1) in terms of its kinetics.

## 4. On the locus of induction of short- and long-term potentiation

As discussed above, the lack of PPF changes during the stable potentiation and the excellent correlation between the inhibition of LTP and the NMDAR-EPSC by the NMDAR-antagonists are most consistent with a postsynaptic mechanism for its induction and expression, for which there is considerable evidence [10,11]. For STP the situation is less straightforward. The finding that STP comprises two components could mean that there are two distinct induction and expression mechanisms for STP(1) and STP(2). In terms of expression, as discussed above, there is evidence from both agonist sensitivity and PPF analysis that STP is presynaptic, or at least can comprise a significant presynaptic component. As discussed above, the lack of correlation between STP(2) and the NMDAR-EPSC in terms of sensitivity to NMDAR-antagonists is compatible with a presynaptic induction. We consider therefore that the simplest explanation for STP, in particular STP(2), is that it is induced and expressed by presynaptic mechanisms, with a trigger being presynaptic NMDARs.

So what is the evidence for the existence of NMDARs on presynaptic terminals of the SCCP? In our first investigation into the effects of the activation of NMDARs in the CA1 region of the hippocampus, we observed a pronounced reduction in the presynaptic fibre volley during, and shortly following, the application of NMDA [9]. This could reflect the presence of presynaptic NMDARs, the activation of which leads to a depolarization of presynaptic elements resulting in the inactivation of sodium channels. However, an alternative explanation is that the activation of postsynaptic NMDARs raises extracellular potassium sufficiently to depolarize the presynaptic terminals, leading to sodium channel inactivation. A third possibility is that the activation of postsynaptic NMDARs helps one to depolarize presynaptic terminals to facilitate the activation of presynaptic NMDARs. In this way, presynaptic and postsynaptic NMDARs would act in unison, with potassium functioning as a retrograde messenger. Whatever the explanation, NMDAR activation can clearly influence presynaptic function. Since our early observation [9], other evidence has accumulated to suggest that there may be NMDARs located presynaptically in the SCCP that can affect conduction of action potentials and neurotransmitter release [26–28]. A role for presynaptic NMDARs in the induction of LTP has also been observed at other central synapses [29].

A particularly interesting discovery was the observation that presynaptic NMDARs can regulate calcium influx into presynaptic boutons in the SCCP and that activation of these receptors is able to increase P(r) [30]. Furthermore, expression of LTP was associated with an increase in P(r), as assessed by using the NMDAR-mediated calcium signals in boutons as an indicator of presynaptic function. These presynaptic NMDARs are sensitive to ifenprodil analogues and so contain GluN2B subunits. We would hypothesize that these presynaptic NMDARs are the ones that trigger STP and, furthermore, that the LTP observed in this study might be that which we term STP(2). More work will, however, be required to verify or refute this hypothesis.

## 5. Can short-term potentiation explain the presynaptic forms of long-term potentiation at CA1 synapses?

Evidence for a presynaptic component for LTP at CA1 synapses comes in many forms [11]. Space does not permit a discussion of all of the papers that have presented evidence for (or against) a presynaptic component for LTP; so, we will restrict our discussion to some of the more pertinent examples. Additional papers that have presented evidence for a presynaptic component of expression of LTP are reviewed in another article in this volume [13].

The initial evidence for a presynaptic component of LTP was obtained at the perforant path input to the dentate gyrus [31,32]. Given the NMDAR-dependence of the LTP induction at the perforant path synapses it is probable that the expression mechanisms of LTP at these synapses are, at least partially, similar to those at CA1 synapses. In these experiments, Bliss and colleagues measured the efflux of radio-labelled or endogenous l-glutamate and used HFS to induce potentiation. They then followed potentiation for an hour or so, during the time when STP is likely to be a major component of potentiation, and found the efflux of glutamate to be increased. These results are therefore consistent with STP being, at least in part, responsible for the increase in l-glutamate release that was observed.

Another approach has been to compare the relative potentiation of the AMPAR- and NMDAR-mediated components of synaptic transmission. This approach has yielded conflicting results. For example, there have been reports of specific potentiation of the AMPAR-mediated component [33], a larger potentiation of the AMPAR-component than of the NMDAR-mediated component [34–36] or a similar potentiation of the two synaptic components at SCCP [37] and perforant path [38] synapses. In addition to observing a similar magnitude of potentiation of the AMPAR- and NMDAR-mediated EPSCs [37], we [39] and others [36,40] observed that the pharmacologically isolated NMDAR-EPSC is able to undergo a substantial LTP, thereby excluding potential interference between the two synaptic components. The conditions of our experiments were such that STP would have dominated these recordings (HFS was used for induction and potentiation was followed by low-frequency stimulation for 30 min). Therefore, these results are consistent with an increase in l-glutamate release contributing to a major component of the potentiation studied during the first 30 min post-induction. These observations can be reconciled with those of the one entirely negative study [33], assuming that LTP does not alter NMDAR function via a postsynaptic mechanism during the time course of their experiments. In these experiments, the failure to observe potentiation of the NMDAR-EPSC may be explained by the use of a pairing protocol (which would not be expected to induce a substantive STP) and prolonged baseline recording before the delivery of HFS (which would prevent LTP from occurring due to washout). Indeed, a subsequent study compared HFS and pairing directly and found a greater potentiation of the NMDAR-EPSC using the former protocol [36]. In summary, results of studies that compared potentiation of the AMPAR- and NMDAR-mediated components of synaptic transmission can be reconciled by the existence of both presynaptic alterations (that lead to enhanced AMPAR and NMDAR-mediated transmission) and postsynaptic alterations (expressed selectively via changes in AMPAR function), the relative contributions of which depend on the experimental conditions employed, particularly, the induction protocol used.

A similar explanation may also apply to the different outcomes of other experiments designed to probe the locus of expression of LTP. For example, the use-dependent NMDAR channel blocker MK-801 was used to compare P(r) in potentiated and control pathways. In the first study, no differences were observed [41]. This study used pairing to induce LTP and performed the MK-801 test over an hour later, so STP would not have contributed to these measurements. In contrast, a second study used HFS, made measurements at a time when a residual STP may have been present and observed a small increase in P(r) in the potentiated input [36].

Complementary techniques have provided evidence that LTP involves an increase in vesicular fusion rates, which is consistent with an increase in P(r). In cultured hippocampal neurons, evidence for an increase in P(r) has been obtained by measuring the rate of vesicle fusion before and after the induction of LTP, using antibody labelling of the synaptic vesicle protein synaptotagmin [42]. Here, the plasticity was induced by application of l-glutamate, which would have access to both presynaptic and postsynaptic NMDARs. The second antibody challenge, used to assess changes in vesicular recycling, commenced 10 min later, at a time when STP would be expected to be a major component of the potentiation. Also, the use of tetrodotoxin to greatly dampen synaptic activity would have prolonged the decay of any STP process. Therefore, this study may have measured the presynaptic changes associated with STP.

In a second approach, the loading of presynaptic boutons with the fluorescent styryl dye FM1-43 was used to assess vesicle fusion rates before and after an NMDAR-LTP induction protocol [43]. An increase in vesicular release was inferred from the experiments, which were followed for up to 1 h. Here, the induction of potentiation was triggered by HFS and only a few stimuli were delivered subsequently (to assess the level of background staining). These conditions are perfect for inducing and sustaining STP and so it is likely that the phenomenon studied in this work is also equivalent to STP in the more intact preparation. An increase in P(r) has also been inferred from FM1-43 experiments in hippocampal slices [44]. Once again, HFS was used to induce the NMDAR-LTP and a low number of test shocks delivered thereafter, conditions that favour STP. Similarly, in a second FM1-43 study in slices, HFS was used to induce LTP and vesicular loading assessed at a time when STP would be prominent [45].

A third method has used hippocampal slices from mice expressing synaptopHluorin [46]. This study identified two temporally distinct components of potentiation and, seemingly contrary to our theory discussed earlier, presented evidence that the presynaptic component of potentiation develops slower than the postsynaptic one. However, the conditions used to monitor vesicular fusion involved a probe test of 50 stimuli at 10 Hz, which may have rapidly eliminated an STP phase of the potentiation. Certainly, very little or no STP was evident in many of the recordings. The possibility of a presynaptic component of expression of LTP (beyond the STP component) is a topic to which we will return to later in this article.

## 6. Interim conclusion

In conclusion, STP and LTP are qualitatively distinct processes. The coexistence but variable expression of the two components of NMDAR-potentiation has, we believe, significantly contributed to the considerably polarized views that are held by some groups regarding the locus of expression of LTP in general. We would argue that groups that have promoted a presynaptic change have, often unwittingly, used stimulus parameters that have favoured STP, while groups that have argued for a postsynaptic expression have, again often unwittingly, employed stimulus parameters that have favoured postsynaptic LTP. However, the separation into STP and LTP is not as simple as the coexistence of two distinct processes. As discussed above, STP comprises two mechanistically distinct components, i.e. STP(1) and STP(2). As discussed below, LTP can also be divided into (at least) two mechanistically distinct components.

## 7. Long-term potentiation can be divided into two mechanistically distinct forms

**Figure 2.**
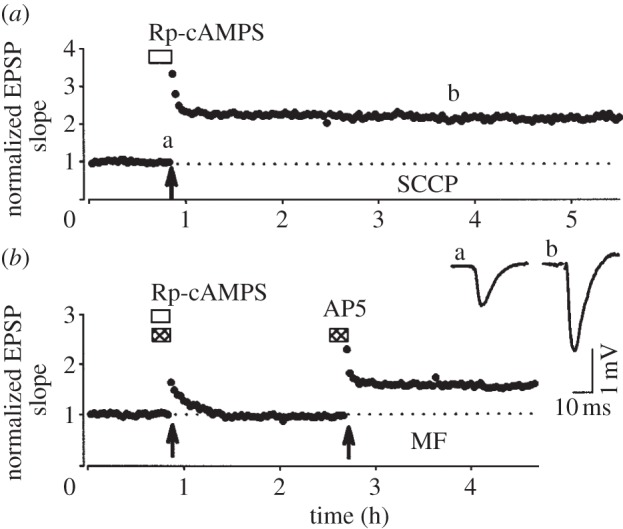
A single HFS can induce a long-lasting PKA-independent form of LTP. (*a*) An example of LTP, induced by a single HFS (100 Hz, 1 s, test intensity) in the presence of a PKA antagonist (Rp-cAMPS; 100 µM). The traces were obtained at the times indicated by (a) and (b). (*b*) In interleaved experiments, the same concentration of Rp-cAMPS was able to fully block the induction of NMDAR-independent LTP in the mossy fibre (MF) pathway. EPSP, excitatory postsynaptic potential. (Adapted from [50] with permission.)

Up until this point we have considered LTP (i.e. the steady increase in synaptic transmission, which follows STP) to be a unitary phenomenon at these CA1 synapses. However, there is considerable evidence that this is not the case. It was discovered that LTP is comprised of an ‘early phase’ that is independent of protein synthesis and a ‘late phase’ that requires de novo protein synthesis for maintenance of LTP; these two components are often referred to as e-LTP (or early-LTP) and l-LTP (late-LTP), respectively. Historically, l-LTP is defined as a form of LTP that requires multiple episodes of HFS for its induction, develops slowly over a period of a few hours, is dependent upon the activation of protein kinase A (PKA) and requires new protein synthesis [47,48]. In these studies, a single episode of HFS was unable to elicit LTP lasting more than 1 h or so [49]. It was suggested that a single tetanus induced a PKA- and protein synthesis-independent form of LTP that was responsible for the early phase of the stable potentiation until l-LTP took over. However, in other studies LTP, lasting many hours, could readily be induced by a single period of HFS in both *in vivo* [7] and *in vitro* [50] experiments (figure 2). Using single (or sometimes multiple) HFS, it has been shown that LTP lasting many hours can be induced at CA1 synapses and that this is neither dependent on the activation of PKA [50] nor does it require new protein synthesis [51–54]. How can these very different observations of NMDAR-LTP at the SCCP be reconciled?

**Figure 3.**
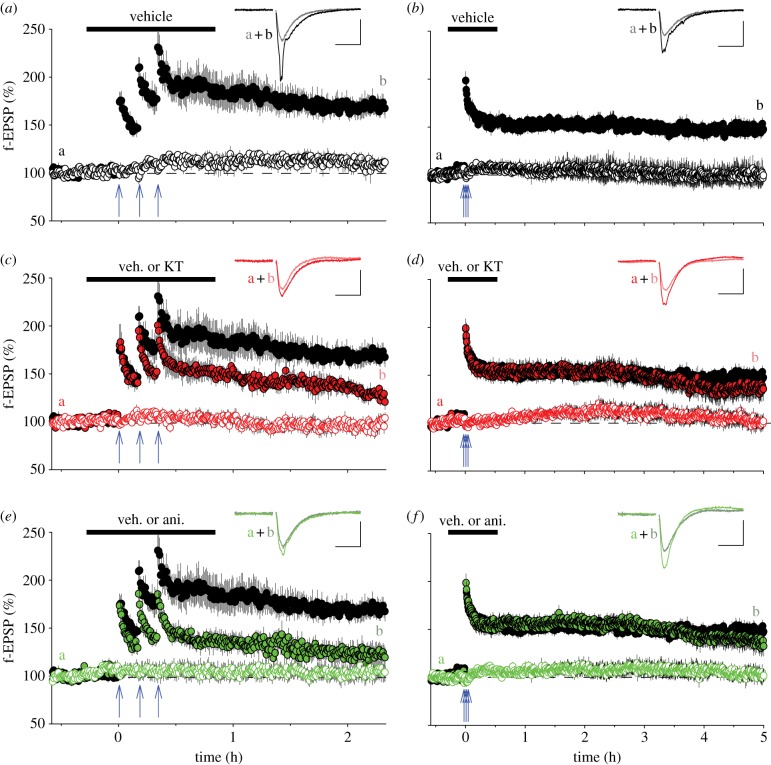
The timing of the induction trigger determines the type of LTP induced. (*a*) LTP induced by spaced HFS (arrows: three trains, 100 Hz, 1 s, test intensity delivered with an inter-train interval of 10 min). The graph plots pooled data for five two-input experiments (filled symbols, test input; open symbols, control input). The inset shows superimposed traces (averages of four successive sweeps; scale bars, 0.5 mV/20 ms in this and the following figure) of baseline and LTP for a typical experiment obtained at the times indicated by (a) and (b). (*b*) LTP induced by compressed HFS (*n* = 6). The protocol was identical to that in (*a*) except that the inter-train interval was 10 s. (*c*) KT 5720 (1 µM) inhibits a component of spaced LTP (*n* = 5). The test and control inputs are shown as filled and open red symbols, respectively, and the control LTP is replotted from (*a*) for ease of comparison. (*d*) KT 5720 (*n* = 7, filled red) has no effect on compressed LTP, which is shown replotted from (*b*). (*e*) Anisomycin (20 µM) inhibits a component of spaced LTP (*n* = 5). The test and control inputs are shown as filled and open green symbols, respectively, and the control LTP is replotted from (*a*). (*f*) Anisomycin (filled green symbols, *n* = 7) has no effect on compressed LTP (replotted from *b*). Data for spaced LTP were quantified 2 h after the delivery of HFS and the levels of LTP were 175±12% of baseline, 127±8% (*p*<0.01) and 124 ± 9% (*p*<0.01) for the vehicle, PKA and anisomycin experiments, respectively. For compressed LTP data were quantified at both 2 and 5 h after HFS. The corresponding values were, after 2 h: 153±4%, 161±6% and 157±8% (*p* > 0.05 both cases; Students *t*-test) and after 5 h: 147 ± 4%, 136 ± 12 and 135 ± 10% (*p* > 0.05 both cases; Students *t*-test). Experiments, which were interleaved in a randomized manner, were performed as described previously [56] with data capture and analysis performed using WinLTP [57]. f-EPSP, field-EPSP.

There are numerous, often unknown, differences in the details of how experiments are performed in various laboratories, any one of which could, in principle, account for properties of the LTP observed. However, all but the experimental variables can be discounted if both forms of LTP can be recorded in the same laboratory under an identical set of conditions. We therefore performed experiments during which the same operator (P.P.), using the same experimental set-up, compared the effects of a single HFS (100 Hz, 1 s) with effects of three identical HFS episodes, separated by 10 min intervals. In agreement with some of the previous reports [49,50,53,55], neither a PKA inhibitor (KT5720) nor a protein synthesis inhibitor (anisomycin) affected the LTP induced by a single HFS (data not shown). In contrast, and also in agreement with some other of the previous observations [47–49,55], these same inhibitors had a substantial effect on the LTP induced by triple HFS (figure 3*c*,*e*). Therefore, the additional LTP that is recruited by a triple HFS can have a different pharmacology to that which is induced by a single HFS. However, the differential induction of these two forms of LTP is not due to the number of stimuli delivered (100 versus 300), but rather depends on the timing of the stimuli. As shown in figure 3*d*,*f*, if the same triple HFS is delivered with only 10 s separating the tetani (compressed HFS) rather than 10 min (spaced HFS) then the resulting LTP is resistant to both KT5720 and anisomycin (at least for the first 5 h following induction). This result confirms a previous report that used KT5720 to compare the effects of four HFS trains with separation intervals of 5 min (spaced) versus effects of tetani with 20 or 3 s separation intervals (compressed) [58] and also confirms the finding that the PKA-independent form of LTP can persist for at least several hours post-induction [50]. In fact, all of these previous data can be reconciled if one simply takes into account the nature of the HFS: single episode or compressed HFS *versus* spaced HFS.

Similar conclusions have also been made using different patterns of TBS [59] and a suggestion has been made that differences in LTP are due to differences in the temporal and spatial characteristics of the levels of cAMP that are generated by compressed and spaced induction protocols. However, differences between spaced and compressed HFS induction protocols have also been observed in mice in which the two Ca^2+^-sensitive adenylyl cyclases (AC1 and AC8) have been knocked out [60]. Therefore, it is unlikely that the induction of PKA-sensitive and insensitive LTP is determined simply by the spatio-temporal characteristics of the cAMP signal.

In terms of protein synthesis, a dependence on the induction protocol has been described previously. It was found that compressed HFS resulted in an LTP that was less sensitive to anisomycin than the LTP induced by a spaced HFS protocol [61]. In some more recent studies, forms of LTP have been described that are completely independent of protein synthesis over a period of at least several hours, whether induced by compressed HFS [51], a single episode of HFS [53] or spaced HFS [53]. In our experiments, we demonstrated both protein synthesis-dependent and independent forms of LTP in interleaved experiments; anisomycin substantially inhibited LTP induced by a spaced HFS while having no effect on LTP induced by a compressed HFS (the latter followed for 5 h).

In summary, our present results verify the existence of two forms of mechanistically distinct LTP (in addition to STP) and show that the only experimental parameter required to selectively elicit one or the other form of LTP in our experimental conditions is the timing of the stimuli within the induction trigger. Two points are clearly evident from these and other studies. First, the PKA- and protein synthesis-independent form of LTP, induced by a compressed HFS, has the capacity to last a very long time (at least 5–6 h, maybe much longer). Second, the PKA and protein synthesis-sensitive component of the LTP, which is induced by spaced stimuli, can develop very quickly. Reduction, due to PKA or protein synthesis inhibition, of this form of LTP is apparent almost immediately following the second HFS stimulus (figure 3*c*,*e*). For this reason, we do not consider that the terms early (i.e. e-LTP) and late (i.e. l-LTP) are appropriate descriptors of these temporally similar processes. Previously, we called these components LTP1 and LTP2, corresponding to the protein synthesis-resistant and sensitive components, respectively [10]. However, this terminology has also been used to subdivide NMDAR-LTP according to other criteria [62,63]. To avoid any confusion, therefore, we refer to these components in the present article as LTPb and LTPc, respectively (reserving LTPa for STP). By this definition, compressed HFS (including a single HFS episode) will induce LTPb whereas a spaced HFS will induce a combination of LTPb and LTPc.

## 8. The locus and mechanisms of expression of LTPb and LTPc

There has been considerable debate as to locus and mechanisms involved in the expression of LTP [11]. Even when one removes the complication of an overlapping STP (as in typical pairing experiments), the situation is far from straightforward. It seems to us to be probable that the existence of two forms of LTP that overlap in time may go some way to addressing some of the controversies. In terms of induction, it is established that both PKA and protein synthesis are required for LTPc but not for LTPb. Although neither KT5720 nor anisomycin are specific for their targets, the findings concerning LTPc have been reproduced using a variety of different PKA and protein synthesis inhibitors and also using various knockouts of PKA subunits [64]. Regarding other kinases, we suspect that the coexistence of LTPb and LTPc may explain some of the controversies in the literature.

In terms of the locus of expression, there is a large body of literature that suggests that LTPb is expressed by postsynaptic mechanisms. Thus, when induction protocols have been employed that we predict would induce LTPb (e.g. a single episode of pairing) the conclusion reached is usually that the LTP is expressed postsynaptically via changes in AMPAR function [11]. There are two (non-exclusive) ways by which AMPAR function can be increased. The first is by altering the efficacy of AMPARs that are already present at the synapse. One mechanism, that could underlie this change is the calcium/calmodulin-dependent protein kinase II (CaMKII)-dependent phosphorylation of GluA1 (on Ser831), which can increase the proportion of time that AMPARs adopt in higher conductance states [65]. The second is by delivering additional AMPARs to synapses [66]. (The new AMPARs may or may not have the same conductance properties as those already at the synapse.) By applying non-stationary fluctuation analysis, which is used to estimate single channel conductance in a population of receptors, to the study of LTP, some of these possibilities have been explored [67,68]. We found examples of LTP that involved no changes in single channel conductance despite large increases in potency, which is most readily explained by an increase in the number of AMPARs of the same conductance that were already present at the synapse. We also found examples of LTP that could be fully explained by an increase in the single channel conductance properties of AMPARs (potentially due to the CaMKII-dependent phosphorylation of GluA1 or by an exchange of higher for lower conductance AMPARs). In addition, we also found examples of a decrease in single channel conductance, which can essentially only be explained by the synaptic incorporation of lower conductance AMPARs. In terms of how AMPARs are recruited into the synaptic plasma membrane, there is evidence for both subunit-dependent [66] and subunit-independent [12] mechanisms, attesting to the likelihood that multiple postsynaptic mechanisms can be drawn upon to increase synaptic strength.

**Figure 4.**
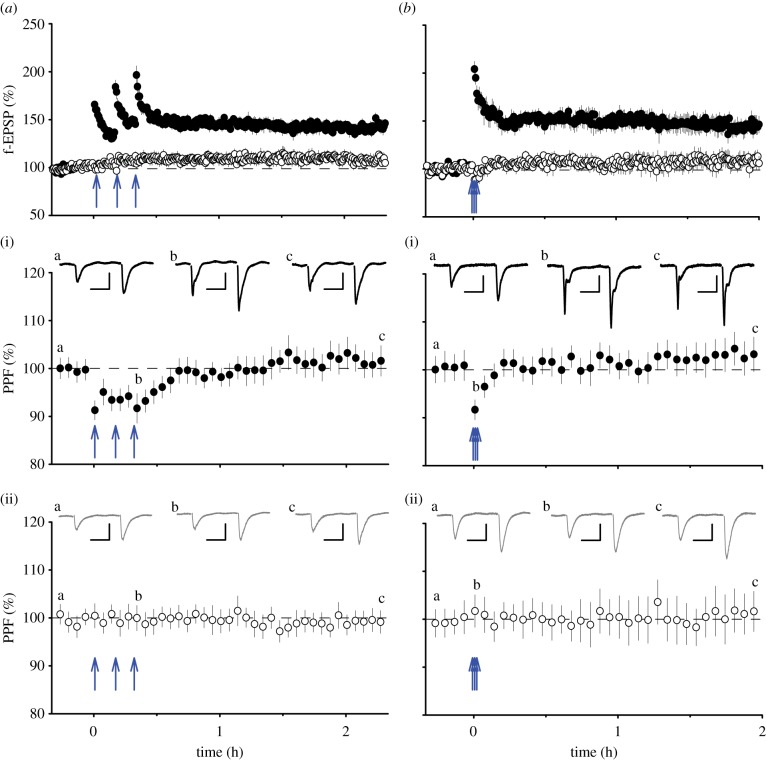
Paired-pulse analysis of LTP. (*a*) LTP induced by spaced HFS (*n* = 6). A plot of paired-pulse ratio (normalized to baseline) for the test (*a*(i), filled circles) and control (*a*(ii), open circles) inputs for these experiments. (*b*) LTP induced by compressed HFS (*n* = 5). A plot of paired-pulse ratio (normalized to baseline) for the test (*b*(i), filled circles) and control (*b*(ii), open circles) inputs for these experiments. There is a reduction in PPF during STP but no significant change thereafter in both sets of experiments. Experiments were performed as described in figure 3, except that paired stimuli (inter-stimulus interval of 50 ms) were delivered throughout the experiment to follow the time course of PPF.

There have been fewer studies regarding the locus of expression of LTPc, as defined by its PKA and protein synthesis dependence. To investigate whether LTPc may involve an increase in P(r) we performed paired-pulse experiments and directly compared LTPc with LTPb. Using both compressed HFS and spaced HFS protocols (to induce either LTPb or LTPb and LTPc, respectively), we observed a decrease in PPF during the STP phase (LTPa) but no alteration in PPF thereafter (figure 4). The fact that we could detect changes during the experiment gives us confidence that the lack of change in PPF during LTPb and/or LTPc is not a technical issue, but rather that it argues against an increase in P(r) during both these forms of potentiation. In apparent contradiction to these findings, a PKA-dependent, presynaptic component of LTP has been described using synaptopHluorin [46]. This component is, however, largely independent of the activation of NMDARs and so may constitute a different form of synaptic plasticity. In other recent work, an NMDAR and protein synthesis-dependent form of LTP has been shown to involve an increase in P(r), based on the use of FM dyes [69]. Thus, both pre and postsynaptic mechanisms may contribute to LTP(c).

**Figure 5.**
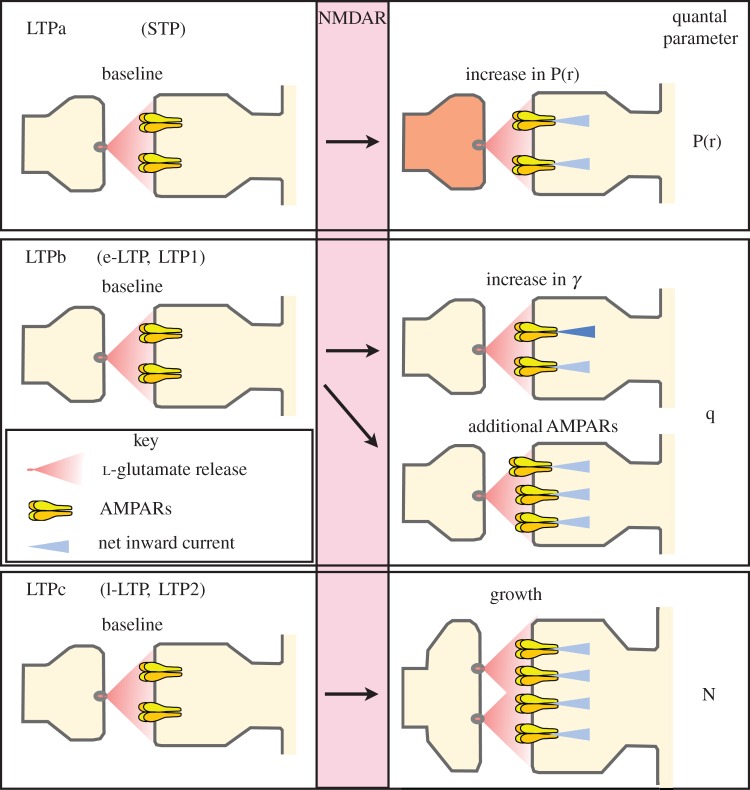
Schematic of different forms of NMDAR-dependent LTP. We suggest that there are multiple forms of LTP that differ in their expression mechanisms. LTPa is characterized by an increase in P(r). It can account for HFS-induced STP (or at least one major component of STP) and possibly for some other forms of LTP. LTPb is characterized by a change in AMPAR function; potentially both as an alteration in their single channel conductance properties (*γ*) and in the number of the receptors. LTPc may be due to synaptic growth, with changes in both the number of release sites (potentially associated with an increase in P(r)) and the number of AMPARs. LTPb corresponds to the PKA and protein synthesis-independent form of LTP (commonly referred to as e-LTP or LTP1 in [10]). LTPc corresponds to the PKA and protein synthesis-dependent form of LTP (commonly referred to as l-LTP or LTP2 in [10]).

There is good evidence that LTP involves a growth of dendritic spines [11]. Potentially, LTPc is the functional correlate of such synaptic growth, with new protein synthesis being required to implement and sustain the structural change. Such a process could involve both an increase in the number of AMPARs at synapses and an increase in the number of functional presynaptic release sites (*N*), possibly associated with an increase in P(r). Potential expression mechanisms for LTPa, LTPb and LTPc are shown schematically in figure 5.

One interesting feature of LTPc is that when it is inhibited by either a PKA inhibitor or a protein synthesis inhibitor the residual LTP is smaller than the level one would expect if LTPb was an entirely independent process. For example, inhibition with anisomycin results in almost complete inhibition of LTPc. Had LTPb been present (the first episode of HFS would induce exclusively LTPb) then a substantial residual LTP would have been expected. One explanation for this phenomenon is that the induction of LTPc leads to the inhibition of the expression of LTPb. This would require a process that was activated during the induction of LTPc and was able to inhibit LTPb even when the expression of LTPc was prevented. For example, activation of a protein phosphatase, in parallel with and independent of PKA, could oppose the actions of kinases involved in LTPb.

## 9. Does long-term potentiation extend beyond a, b, c?

Our discussions of LTP have been restricted to the first few hours following its induction. During this period, LTP may, or may not, also be dependent upon transcription [70]. In some studies, a transcriptional-dependent phase of LTP has been defined based on sensitivity to actinomycin D [71]. Tentatively this could constitute an additional component of LTP, which according to the present nomenclature would be termed LTPd.

As far as the first three components of LTP are concerned, some outstanding questions remain. We define LTPa as a component that is expressed as an increase in P(r) and we equate it to STP, which is observed as a decaying component of synaptic potentiation in many slice experiments. However, we have shown that STP can itself be divided into two kinetically and pharmacologically distinct processes, which we have defined as STP(1) and STP(2). Further work is required to establish precisely how these two components of STP relate to LTPa. Regarding LTPb, we have defined this on the basis of its resistance to inhibitors of PKA and protein synthesis. We feel that there is overwhelming evidence that this comprises, at least in major part, a postsynaptic alteration. However, we do not necessarily consider that it can be explained by a single postsynaptic mechanism, but rather it might involve alterations in AMPAR trafficking and changes in AMPAR single channel conductance properties. In which case, LTPb may need to be further subdivided (LTPb1, LTPb2, etc.) according to mechanistic criteria. We have defined LTPc on the basis of its dependence on PKA and protein synthesis. Less is known concerning its expression mechanisms, though we speculate that this could involve both presynaptic and postsynaptic changes, and hence might also require a subcategorization.

The situation becomes even more complex when one considers that plasticity mechanisms alter throughout the lifespan of an animal. For example, ‘silent synapses’, defined as synapses that lack functional AMPARs [72,73], are especially pronounced early in development [74]. LTP can involve ‘unsilencing’ of these synapses by a mechanism that is generally considered to involve the insertion of AMPARs. Once AMPARs are present at synapses, a variety of additional mechanisms can be recruited to increase synaptic strength. These include processes that are most simply explained by an increase in P(r) [68,75] as well as mechanisms that probably involve alterations in both AMPAR trafficking and single channel properties [68]. The multitude of expression mechanisms is exemplified in this latter study, which identified two very distinct LTP phenotypes in slices from 1-week-old rats and two further distinct LTP phenotypes in slices from 2-week-old rats. How these developmental and mature forms of LTP relate to one another mechanistically is largely unknown. The family of LTP mechanisms is, of course, further diversified by the existence of NMDAR-independent forms of LTP [76], including those triggered by the synaptic activation of kainate receptors [77], calcium-permeable AMPARs [78] and voltage-gated Ca^2+^ channels [46].

A challenge for the future is to establish why multiple tetani are effective at inducing LTPc when spaced at 10 min but not 10 s intervals. One possibility is that at short intervals there is an inhibitory influence of a synaptic depression induced by the first tetanus that interferes with the subsequent tetani to prevent induction of LTPc. Another possibility is that the first tetanus ‘primes’ the synapse to enable subsequent tetani to induce LTPc. In which case, establishing the ‘priming’ mechanism will be a key issue.

## 10. Concluding remarks

We have articulated here the view that NMDAR-LTP at CA1 synapses in the hippocampus is not a uniform process but comprises a family of plasticity mechanisms. We believe that the coexistence of these different forms of LTP may explain, to a large extent, many of the controversies that have plagued the field for far too long. We have described how the different forms of LTP depend on the stimulus parameters that are used to trigger the process. Our conclusion that HFS favours LTPa and that this is expressed by an increase in P(r) is consistent with the conclusion reached in another article in this volume that HFS favours presynaptic LTP [13]. Our study goes further in two respects: firstly, it divides LTP into more than two components. Secondly, it defines more precisely the stimulation requirements for the induction of the different forms of LTP (HFS for LTPa, spaced stimulation for LTPc). In the other article [13] it is argued that the reason why HFS induces presynaptic LTP is because HFS leads to an increased postsynaptic depolarization that recruits L-type voltage-gated Ca^2+^ channels in the postsynaptic cell; this then triggers the release of NO that acts as a retrograde messenger to convey the induction signal to the presynaptic terminal. We have not considered the relative roles of L-type Ca^2+^ channels or retrograde messengers. For LTPa we have suggested that both the induction and the expression may be presynaptic in origin, although additional postsynaptic factors cannot be ruled out. For LTPc, a process that we speculate has both presynaptic and postsynaptic components, the existence of retrograde signalling is not incompatible with any of the evidence that we have assessed.

Our conclusions are also compatible with the views expressed by the authors of another article in this volume [12], provided one equates their LTP with LTPb. They define the conditions for their study of LTP, such that they can ‘ignore’ modulatory influences and focus directly on expression mechanisms. As described above, such conditions mitigate against induction of LTPa and, we suspect based on the properties of the LTP they describe, also mitigate against LTPc. We argue therefore that these authors are correct in their conclusions, with respect to the locus of expression of their LTP, but that they are only considering one possible type of LTP. They rightly highlight the importance of the agonist uncaging experiments that reveal that a postsynaptic modification can occur during this form of LTP, a conclusion that we had reached many years earlier using the localized ionophoretic application of AMPAR ligands [17]. In our study, the increase in sensitivity correlated with the generation of what we define here as LTPb.

The fact that multiple forms of LTP can be simultaneously triggered at the same type of synapse greatly increases the functional utility of this family of processes. For example, the responses evoked by a high-frequency input will be differentially potentiated during LTPa but will be uniformly enhanced during LTPb and/or LTPc. Furthermore, the activity-dependent decay of LTPa could endow synapses with the property to store information until it is accessed, a potential synaptic correlate of working memory. Even in its most commonly observed form, as a rapidly decaying form of plasticity, LTPa could represent the most widely used form of synaptic plasticity in the brain, since far more information is stored for short periods of time than is committed to long-term storage. While further work is, of course, needed to understand the full functional significance of various forms of NMDAR-LTP, we are of the firm opinion that these can serve multiple cognitive functions and that the beauty of the process lies in its diversity.
